# Vitamin D for type 2 diabetes prevention: cost-effective, but not yet cost-saving

**DOI:** 10.1016/j.lana.2026.101628

**Published:** 2026-09-05

**Authors:** Jiafeng Zhang, Xinhui Zhan, Tuo Li

**Affiliations:** a Department of Endocrinology, No. 905 Hospital of PLA Navy, Shanghai Changzheng Hospital, Naval Medical University, Shanghai, 200052, China; b Department of Endocrinology, Shanghai Changzheng Hospital, Naval Medical University, Shanghai, 200003, China

Briody and colleagues provide the first cost-effectiveness analysis of vitamin D for preventing type 2 diabetes in US adults with prediabetes, addressing a gap identified by the 2024 Endocrine Society guideline.^1^ The microsimulation is careful and transparent, and the conclusion that supplementation is economically attractive is plausible. The headline that vitamin D is “cost-saving,” however, rests on features of the model's own uncertainty that deserve a closer look.

A cost-saving (dominant) intervention must both improve health and reduce net cost. Over the lifetime the incremental-cost interval spans −$5974 to +$171 and the ICER interval −$72,486 to +$3947—both include zero.^1^ Net monetary benefit is firmly positive ($8108–$21,907), so supplementation is cost-effective over a lifetime; dominance is less secure. The authors report that 99% of probabilistic iterations were cost-saving, but this too is a lifetime estimate, and the base-case interval still admits a net cost increase. “Cost-saving” and “cost-effective” carry different weight for guideline strength, formulary decisions, and population-scale savings claims.

The favourable result is generated largely beyond the evidence. The source trials had a median follow-up near three years,^2^ yet roughly 87–99% of the modelled lifetime benefit accrues after year 10 (Figure S1 in the Supplementary Material). At the 10-year horizon—closest to the trial data—incremental life-years, QALYs, cost, and net monetary benefit all have intervals that cross zero. The authors’ shorter-horizon and waning-efficacy scenarios stay nominally positive, but only as point estimates without reported uncertainty. The lifetime precision therefore depends on assuming a 15% risk reduction sustained undiminished across decades, not on what the trials observed.

The 15% effect is itself pooled across heterogeneous agents: two trials used cholecalciferol and one used the active analogue eldecalcitol, whose non-significant result (hazard ratio 0.87)^3^ and distinct pharmacology and hypercalcaemia profile differ from the 4000 IU/day cholecalciferol modelled here. Applying the combined estimate to a single nutritional dose adds structural uncertainty the simulation does not propagate.

None of this overturns the study's contribution; vitamin D may well be a cost-effective addition to prevention. We suggest the conclusion be stated as potentially cost-effective rather than cost-saving, and that horizon-specific uncertainty be reported alongside the lifetime point estimate.^4^ Precision the model cannot support risks over-promising to guideline panels and payers.

## Contributors

JZ: writing–original draft, writing–review & editing, data analysis, validation, visualisation, supervision. XZ: literature search, investigation, writing–review & editing. TL: writing–review & editing, supervision. All authors had full access to all the data in the study, and the corresponding author had final responsibility for the decision to submit for publication.

## Declaration of interests

JZ, XZ, and TL declare no competing interests.
