# Supplementary material for: Vitamin D for type 2 diabetes prevention: cost-effective, but not yet cost-saving
Source: Lancet Reg Health Am. 2026 Sep 5;62:101628. doi: 10.1016/j.lana.2026.101628 (PMC13571935; doi:10.1016/j.lana.2026.101628)
Supplement: Supplementary file 1 [file mmc1.pdf]

Supplementary Material

Vitamin D for type 2 diabetes prevention: cost-effective, but not yet cost-saving

Manuscript reference: TLRHAMERICAS-D-26-00741

Jiafeng Zhang, Xinhui Zhan, Tuo Li

Contents

Supplementary Figure. Figure 1 and legend....2

Supplementary Methods. Re-analysis of the reported cost-effectiveness estimates of Briody et al....3

Table S1. Incremental outcomes extracted from Briody et al....3

Appendix. Calculation script (Python)....5

Supplementary Figure

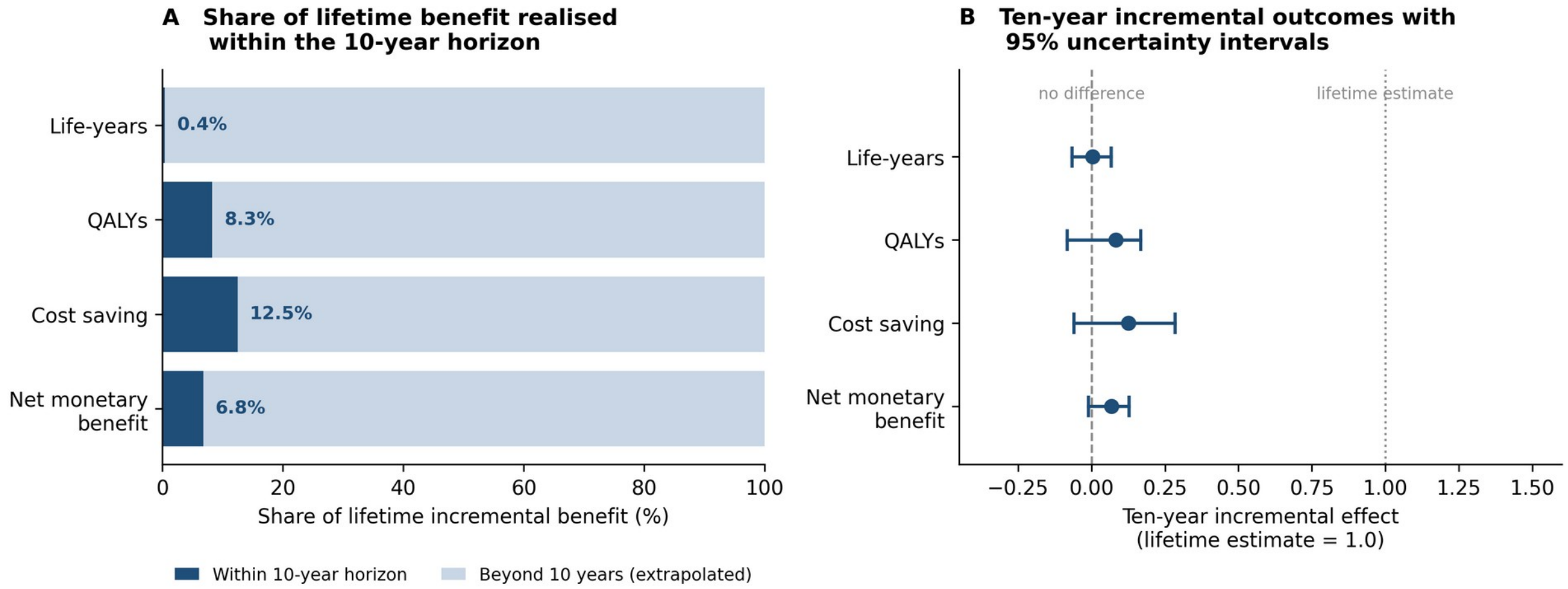

Figure 1. Re-analysis of Briody et al. (Table 3) using the authors' own point estimates and 95% uncertainty intervals (UIs). (A) Proportion of the lifetime incremental benefit realised within the 10-year horizon; approximately 87–99% of each modelled benefit accrues after year 10. (B) Ten-year incremental outcomes, each normalised to its corresponding lifetime point estimate (=1.0), with 95% UIs; all four intervals include zero. Normalisation is for display only and does not alter which intervals cross zero.

Supplementary Methods: Re-analysis of the reported cost-effectiveness estimates of Briody et al.

1. Objective

The published analysis reports vitamin D supplementation as "cost-saving" over a lifetime horizon. Our objective was to characterise, using the authors' own reported point estimates and 95% uncertainty intervals (UIs), (i) whether each incremental outcome is statistically distinguishable from no difference, (ii) how much of the lifetime benefit is realised within the horizon that is closest to the underlying trial evidence, and (iii) the appropriate wording of the economic conclusion. This is a transparency re-analysis of already-published summary results; it introduces no new model, parameters, or data.

2. Data source and extraction

All inputs were extracted verbatim from Briody et al.: incremental remaining life-years, discounted costs, discounted QALYs, the net monetary benefit (NMB) and the incremental cost-effectiveness ratio (ICER), each for the 10-year and lifetime horizons, together with their 95% UIs (Table 3; the ICER UI is also given in Table 4). As reported by the authors, point estimates are the mean across 100 model runs of 10,000 simulated individuals, and each 95% UI is the 2.5th–97.5th empirical percentile of the across-run distribution. NMB is defined by the authors as (QALYs gained × $100,000) − incremental cost. The extracted values are reproduced in Table S1; we introduced no rounding beyond that already present in the source table.

Table S1. Incremental outcomes extracted from Briody et al. (intervention minus no-intervention). Costs are discounted; a negative incremental cost is a saving. "UI incl. 0" indicates whether the reported 95% UI contains zero.

Outcome	10-yr incremental (95% UI)	incl. 0?	Lifetime incremental (95% UI)	incl. 0?	%≤10 yr
Remaining life-years	0.001 (−0.018 to 0.018)	Yes	0.27 (0.06 to 0.50)	No	0.4%
Discounted cost, $	−401 (−908 to 192)	Yes	−3208 (−5974 to 171)	Yes	12.5%
Discounted QALYs	0.01 (−0.01 to 0.02)	Yes	0.12 (0.05 to 0.19)	No	8.3%
Net monetary benefit, $	1058 (−172 to 1964)	Yes	15,483 (8108 to 21,907)	No	6.8%
ICER, $/QALY	−61,122 (−1,033,808 to 1,005,279)	Yes	−26,134 (−72,486 to 3947)	Yes	—

3. Analysis 1 — Compatibility of each incremental outcome with no difference

For every incremental outcome at each horizon we recorded whether the authors' reported 95% UI contained zero. Because these UIs are the model's own uncertainty, an interval spanning zero indicates that the direction of the incremental effect is not resolved under the model's parameterisation, i.e. the result is compatible with no benefit. At the 10-year horizon—closest to the ~3-year median follow-up of the source trials—all five metrics (life-years, cost, QALYs, NMB, ICER) have UIs that include zero. At the lifetime horizon, life-years, QALYs and NMB exclude zero, but the incremental cost (−$5974 to + $171) and the ICER (−$72,486 to +$3947) still include zero.

This separates two claims that are often conflated. Cost-effectiveness requires a positive NMB; by this criterion the intervention is supported at the lifetime horizon (NMB UI $8108–$21,907, entirely positive) but not at 10 years (NMB UI −$172 to $1964). A cost-saving (dominant) intervention additionally requires the incremental cost to be negative with an interval excluding zero; this stronger condition is met at neither horizon. The authors' probabilistic sensitivity analysis found dominance in 99% of iterations, but that figure is a lifetime result obtained under the same multi-decade extrapolation (Analysis 2), and it does not reconcile with the base-case interval for incremental cost, which admits a net increase. We therefore treat cost-saving as probable but not established with the certainty an unqualified label implies, whereas lifetime cost-effectiveness is well supported.

4. Analysis 2 — Share of lifetime benefit realised within the evidence-proximate horizon

For each additive outcome we computed the fraction of the lifetime incremental estimate already realised by year 10 as: share = (10-year incremental point estimate) ÷ (lifetime incremental point estimate) × 100%. This yields 0.4% for life-years, 8.3% for QALYs, 12.5% for cost savings and 6.8% for NMB (Table S1; Figure 1A). Equivalently, roughly 87–99% of every modelled benefit accrues after year 10—beyond any horizon supported by trial data and under the base-case assumption that a 15% relative risk reduction persists undiminished for decades. The lower end of this range (≈87–93%) is anchored by the cost and NMB shares, which are reported to full dollar precision; the life-year and QALY shares are less precise because their 10-year point estimates are reported to few decimals. The ICER was not decomposed this way because it is a ratio, not an additive quantity.

5. Analysis 3 — Normalisation for Figure 1B

To display outcomes measured in different units (years, QALYs, dollars) on a common axis, each 10-year incremental estimate and its 95% UI were divided by the corresponding lifetime incremental point estimate, so that the lifetime effect maps to 1.0 and no difference maps to 0. Cost is plotted as a saving (sign reversed, so positive denotes a saving). This is a rescaling for visualisation only; it is monotonic and does not change which intervals include zero. Figure 1B thus shows, on a single scale, that all four 10-year incremental outcomes sit near zero with UIs crossing it, while the lifetime reference is 1.0.

6. Descriptive contextualisation of the lifetime QALY gain

To convey scale, the lifetime incremental gain of 0.12 QALYs was expressed as perfect-health-equivalent time: 0.12 × 365 ≈ 44 days accrued over the intervention arm's ~36.7 remaining life-years, i.e. about 1.2 days per person-year. This is a descriptive conversion and was not used in any inferential step.

7. Software and reproducibility

All computations were performed in Python 3.11 (pandas 2.3); the Figure was produced with Matplotlib. No proprietary software, licence, or individual-level data are required: every value is a deterministic function of the summary estimates in Briody et al. Table 3/Table 4. The complete calculation is given in the Appendix and can be re-run in seconds.

8. Assumptions and limitations of this re-analysis

First, we used the reported summary estimates and treated each 95% UI as published; we did not have access to run-level outputs, so the zero-crossing assessment reflects the authors' own intervals rather than an independent recomputation. Second, "includes zero" is a compatibility statement appropriate to a

simulation-based uncertainty interval, not a formal frequentist hypothesis test. Third, the share-of-benefit ratios in Analysis 2 use point estimates only and do not propagate uncertainty; the 10-year life-year and QALY point estimates are reported to limited decimal precision, so those shares are approximate. Fourth, the normalisation in Analysis 3 affects display only. Finally, we did not attempt to reproduce or alter the microsimulation itself; the aim was to characterise, not to recompute, the published outputs. None of these limitations affects the two central, directly verifiable observations: the reported incremental-cost interval includes zero at both horizons, and the great majority of the modelled benefit accrues after the evidence-proximate 10-year horizon.

9. Data availability

All source numbers are contained in Table 3 and Table 4 of Briody et al. (Lancet Reg Health Am 2026;61:101543). The extracted values (Table S1) and the calculation script (Appendix) are provided so that every figure value can be reproduced from the published article alone.

Appendix. Calculation script (Python)

```
import pandas as pd
# (est, lo, hi): incremental estimate and 95% UI, from Briody et al. Table 3/4
d = {
  'life_years': {'t10': (0.001,-0.018,0.018), 'life': (0.27,0.06,0.50)},
  'cost'      : {'t10': (-401,-908,192),       'life': (-3208,-5974,171)},
  'QALYs'     : {'t10': (0.01,-0.01,0.02),     'life': (0.12,0.05,0.19)},
  'NMB'       : {'t10': (1058,-172,1964),      'life': (15483,8108,21907)},
  'ICER'      : {'t10': (-61122,-1033808,1005279),'life': (-26134,-72486,3947)},
}
incl0  = lambda lo,hi: lo <= 0 <= hi           # 95% UI includes zero?
share  = lambda a,b: 100*a/b                    # % of lifetime realised by yr 10
for k,v in d.items():
    e10,l10,h10 = v['t10']; eL,lL,hL = v['life']
    print(k, 'UI incl 0 (10y):', incl0(l10,h10),
             '| UI incl 0 (life):', incl0(lL,hL),
             '| %<=10y:', round(share(e10,eL),1) if k!='ICER' else 'NA')
print('QALY 0.12 ->', round(0.12*365,1), 'days over ~36.7 life-years')
```
